# Cortisol Circadian Rhythm and Sarcopenia in Patients With Type 2 Diabetes: A Cross‐Sectional Study

**DOI:** 10.1002/jcsm.13727

**Published:** 2025-02-17

**Authors:** Fupeng Liu, Qing Yang, Kai Yang, Jing Sun, Yanying Li, Bo Ban, Yangang Wang, Mei Zhang

**Affiliations:** ^1^ Department of Endocrinology, Affiliated Hospital of Jining Medical University Jining Medical University Jining China; ^2^ Department of Clinical Nutrition, Affiliated Hospital of Jining Medical University Jining Medical University Jining China; ^3^ Department of Clinical Medicine Jining Medical University Jining China; ^4^ Department of Endocrinology, Affiliated Hospital of Medical College Qingdao University Qingdao China

**Keywords:** cortisol circadian rhythm, muscle mass, muscle quality, sarcopenia, Type 2 diabetes

## Abstract

**Background:**

Patients with Type 2 diabetes mellitus (T2DM) have elevated late‐night cortisol levels and a flattened circadian rhythm. Cortisol oversecretion mediates muscle breakdown and reduces muscle strength and mass, thus possibly leading to sarcopenia. This study first investigated the association between cortisol circadian rhythm and sarcopenia in patients with T2DM.

**Methods:**

Patients with T2DM and adrenal nodules were screened for eligibility. Skeletal muscle index (SMI) and skeletal muscle density (SMD) were obtained by analysing computed tomography images at Lumbar 3 level. Sarcopenia was defined as the presence of both myopenia and myosteatosis. Cortisol and adrenocorticotropic hormone levels at 8 AM, 4 PM and 0 AM were measured. The cumulative logit models and receiver operating characteristic (ROC) curve analyses were performed to evaluate the association between cortisol circadian rhythm and sarcopenia.

**Results:**

In total, 128 patients with T2DM and nonfunctional adrenal adenomas were enrolled in this study, of whom 25 were diagnosed with sarcopenia. The mean age was 54.4 years, and 83 (64.8%) patients were male. Patients with sarcopenia showed higher nighttime cortisol levels at 0 AM (Cor 0 AM) (4.91 [4.05, 9.95] vs. 2.44 [1.55, 4.77] μg/dL, *p* < 0.001) than those without. The Cor 0 AM was negatively correlated with both SMI and SMD (*r* = −0.318, *p* < 0.001 and −0.284, *p* < 0.001, respectively). As the Cor 0 AM tertiles increased, the odds ratios (ORs) for sarcopenia consistently increased (OR = 4.69 [0.93, 23.53], *p* = 0.061, for the intermediate group and OR = 11.39 [2.41, 53.84], *p* = 0.002, for the high group). After adjustment for multiple risk factors, the high Cor 0 AM group still showed a significantly higher risk of sarcopenia than the low group (OR = 7.92 [1.45, 43.29], *p* = 0.017). ROC curve analyses showed that Cor 0 AM had the highest predictive power for sarcopenia, with an area under the ROC curve (AUC) of 0.760, compared to haemoglobin, age, alanine transaminase and sex (AUC = 0.703, 0.695, 0.679, and 0.633, respectively).

**Conclusions:**

The cortisol circadian rhythm is associated with sarcopenia in patients with T2DM. Patients with higher levels of nighttime cortisol, rather than morning or afternoon cortisol, have a higher risk of sarcopenia. This result offers a new strategy for the further research of sarcopenia.

## 1 Introduction

Type 2 diabetes mellitus (T2DM) is a major public health crisis globally [1]. Approximately 537 million adults worldwide had diabetes in 2021, most of whom had Type 2 diabetes, and this number is expected to rise to 783 million by 2045 [2]. Apart from its common complications, patients with T2DM have a higher risk of sarcopenia, which heavily impacts quality of life and increases risk of fracture, disability and mortality. Sarcopenia is emerging as a third category of diabetic complication [3, 4, 5].

The high prevalence of sarcopenia in patients with T2DM may be due to diabetes‐related risk factors such as insulin resistance, inflammation, accumulation of advanced glycation end‐products, increased oxidative stress and vascular complications [4, 5, 6]. Moreover, endocrine changes, particularly with cortisol, also seem to be crucial in the development of sarcopenia [7]. The oversecretion of cortisol mediates muscle breakdown, reduces muscle strength and mass and thus possibly leads to sarcopenia [8, 9]. Few of the studies have demonstrated that patients with T2DM have elevated late‐night cortisol levels and a flattened circadian rhythm [8, 10, 11]. However, the association between the cortisol rhythm and sarcopenia in patients with T2DM and the general population has not yet been clarified.

Sarcopenia is defined as the presence of low muscle mass and reduced muscle quality in strength or performance [12]. Computed tomography (CT) is considered the gold standard for the noninvasive assessment of muscle health, which can be used for its diagnosis [12, 13, 14]. In this study, we aimed to investigate whether cortisol rhythm is associated with sarcopenia in patients with T2DM. In here, we defined sarcopenia as the presence of both myopenia and myosteatosis (reduced muscle quality) based on abdominal CT scans [15, 16].

## 2 Methods

### 2.1 Study Population

Patients with T2DM and adrenal nodules hospitalized in the Department of Endocrinology, Affiliated Hospital of Jining Medical University from January 2017 to December 2021, were screened for eligibility. To evaluate the characteristics and functionality of these nodules, abdominal CT scans and cortisol circadian rhythm analyses were conducted. The cortisol circadian rhythm analysis measured cortisol and adrenocorticotropic hormone (ACTH) levels at 8 AM, 4 PM and 0 AM. And the patients diagnosed with nonfunctional adrenal adenomas were included in the study. The exclusion criteria were as follows: (1) patients diagnosed with functioning adrenal adenoma, pheochromocytoma or adrenal carcinoma; (2) missing major anthropometric measures such as height and weight; (3) having malignant tumours, Stage V diabetic nephropathy, immune diseases or pituitary diseases; (4) having acute complications or infections; and (5) receiving corticosteroids. All patients were informed that their medical records from their hospital stay could be used for scientific research, and they provided written broad consent. This study was approved by the Ethics Committee of the Affiliated Hospital of Jining Medical University (No. 2021C041).

### 2.2 Laboratory and Body Composition Measurements

All biochemical and immune indices were measured at our hospital laboratory. Cortisol and ACTH levels were measured using chemiluminescence assay kits (Roche Diagnostics, Mannheim, Germany). Body composition was measured as our previous study [17]. Two authors identified axial CT images at the L3 level, in which the spinous process and two transverse processes could be visualized. Slice‐O‐Matic software (V.5.0, TomoVision, Montreal, Quebec, Canada) was used for image analysis with attenuation thresholds from −29 to 150 Hounsfield units (HU) for skeletal muscle, −150 to −30 HU for visceral adipose tissue and −190 to −30 HU for subcutaneous adipose tissue. Data outputs were provided as area in squared centimetres and CT attenuation for each tissue type. The skeletal muscle index (SMI) and fat mass index (FMI), which reflect muscle and adipose mass, respectively, were calculated by normalizing the skeletal muscle or adipose (visceral plus subcutaneous) area in squared centimetres by height in squared metres. Skeletal muscle density (SMD), which reflects muscle quality, was determined as the mean CT attenuation. The criteria for myopenia were SMI < 52·4 cm^2^/m^2^ for men and < 38·5 cm^2^/m^2^ for women [15]. The criteria for myosteatosis were SMD < 33 HU with BMI ≥ 25 kg/m^2^ or SMD < 41 HU with BMI < 25 kg/m^2^ [16]. Sarcopenia was defined as the presence of both myopenia and myosteatosis.

### 2.3 Statistical Analysis

Continuous variables were shown as median and interquartile range if not normally distributed and as mean ± SD if normally distributed. Categorical variables were summarized as frequency counts and percentages. Characteristics of the study population were compared according to sarcopenia categories and cortisol tertiles. Continuous variables were compared using an independent samples *t* test, Mann–Whitney *U* test, one‐way analysis of variance (ANOVA) or Kruskal–Wallis test. Categorical variables were analysed using the *χ*2 test. Univariable correlations between age, cortisol, ACTH and body composition variables were assessed using Spearman's correlation coefficients. To assess the independent associations between cortisol tertiles and sarcopenia, a cumulative logit model was used to obtain unadjusted and adjusted odds ratios (ORs) for the likelihood of sarcopenia. The following adjustment models were applied: Model 1 was adjusted for variables with significant differences between the control and sarcopenia groups; Model 2 was further adjusted for all the variables included in this study. Receiver operating characteristic (ROC) curve analyses were used to compare the diagnostic performance of risk factors for sarcopenia according to the area under the ROC curve (AUC). Statistical analyses were performed using SPSS software (Version 27.0; IBM Corp., Armonk, NY, USA). A two‐tailed statistical measure with a *p* value less than 0.05 was considered significant.

## 3 Results

### 3.1 Clinical Characteristics of Enrolled Patients

**FIGURE 1:**
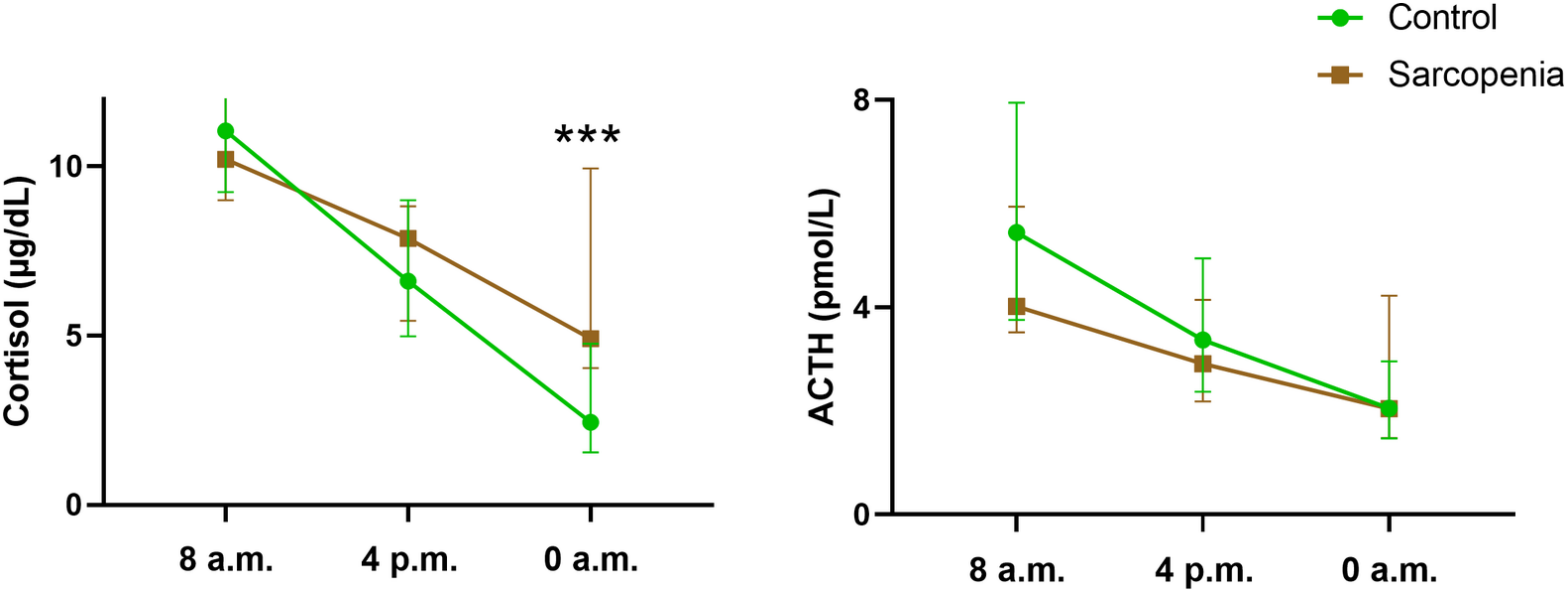
Cortisol and ACTH circadian rhythm in control and sarcopenia group. ***p < 0.001.

In total, 128 patients were enrolled in this study, of whom 25 were diagnosed with sarcopenia. The mean age was 54.4 years, and 83 (64.8%) patients were male. Body composition, cortisol rhythm and clinical characteristics are shown in Table 1. Patients with sarcopenia showed higher levels of cortisol at 0 AM (Cor 0 AM) (4.91 [4.05, 9.95] vs. 2.44 [1.55, 4.77] μg/dL, *p* < 0.001) and age and lower male percentage, haemoglobin, alanine transaminase, SMI and SMD compared with control group. The circadian rhythms of the cortisol and ACTH levels in the control and sarcopenia groups are shown in Figure 1.

**TABLE 1:** Clinical characteristics of included patients.

Variables	Control group (*n* = 103)	Sarcopenia group (*n* = 25)	*p* value
Male (%)	72 (69.9)	11 (44.0)	0.020
Age (years)	52.66 ± 13.82	61.72 ± 9.24	< 0.001
DM course (years)	6.00 (2.00, 13.00)	10.00 (4.00, 16.50)	0.111
BMI (kg/m^2^)	26.35 ± 3.57	25.27 ± 2.89	0.161
Haemoglobin (g/L)	143.43 ± 15.57	128.28 ± 31.10	< 0.001
Creatinine (μmol/L)	62.57 ± 15.06	56.46 ± 14.68	0.071
Blood urea nitrogen (mmol/L)	5.48 ± 1.45	5.41 ± 1.93	0.855
TG (mmol/L)	1.37 (0.92, 2.18)	1.33 (0.91, 1.82)	0.605
Total cholesterol (mmol/L)	4.68 ± 1.21	4.57 ± 1.14	0.665
HDL (mmol/L)	1.16 ± 0.25	1.21 ± 0.25	0.350
LDL (mmol/L)	2.83 ± 0.86	2.85 ± 1.05	0.898
Albumin (g/L)	44.24 ± 4.22	42.34 ± 5.42	0.060
Alanine transaminase (U/L)	17.50 (13.98, 30.53)	12.70 (10.80, 18.65)	0.006
FBG (mmol/L)	7.64 ± 2.60	7.92 ± 3.39	0.643
HbA1c (%)	8.74 ± 2.03	9.07 ± 3.26	0.625
SBP (mmHg)	138.00 ± 19.28	147.12 ± 28.53	0.140
DBP (mmHg)	83.95 ± 17.80	82.20 ± 16.68	0.656
Dyslipidemia (%)	60 (58.3)	11 (44.0)	0.262
Hypertension (%)	66 (64.1)	17 (68.0)	0.817
Cigarette smoking (%)	40 (38.8)	8 (32.0)	0.647
Alcohol intake (%)	44 (42.7)	8 (32.0)	0.371
SMI (cm^2^/m^2^)	49.85 ± 9.44	37.96 ± 7.18	< 0.001
SMD (HU)	38.97 ± 6.59	28.43 ± 3.68	< 0.001
FMI (cm^2^/m^2^)	107.95 ± 40.76	110.64 ± 27.68	0.754
Cor 8 AM (μg/dL)	11.05 (9.24, 14.00)	10.21 (9.00, 16.00)	0.831
Cor 4 PM (μg/dL)	6.61 (4.98, 9.00)	7.87 (5.45, 8.82)	0.412
Cor 0 AM (μg/dL)	2.44 (1.55, 4.77)	4.91 (4.05, 9.95)	< 0.001
ACTH 8 AM (pmol/L)	5.44 (3.76, 7.95)	4.02 (3.52, 5.94)	0.071
ACTH 4 PM (pmol/L)	3.37 (2.37, 4.94)	2.91 (2.18, 4.14)	0.253
ACTH 0 AM (pmol/L)	2.05 (1.47, 2.96)	2.04 (1.48, 4.22)	0.387

Abbreviations: Cor, cortisol; DBP, diastolic blood pressure; DM, diabetes mellitus; FBG, fasting blood glucose; HDL, high‐density lipoproteins; LDL, low‐density lipoproteins; SBP, systolic blood pressure; TG, triglycerides.

### 3.2 Correlations Between Body Composition, Age and Cortisol

**FIGURE 2:**
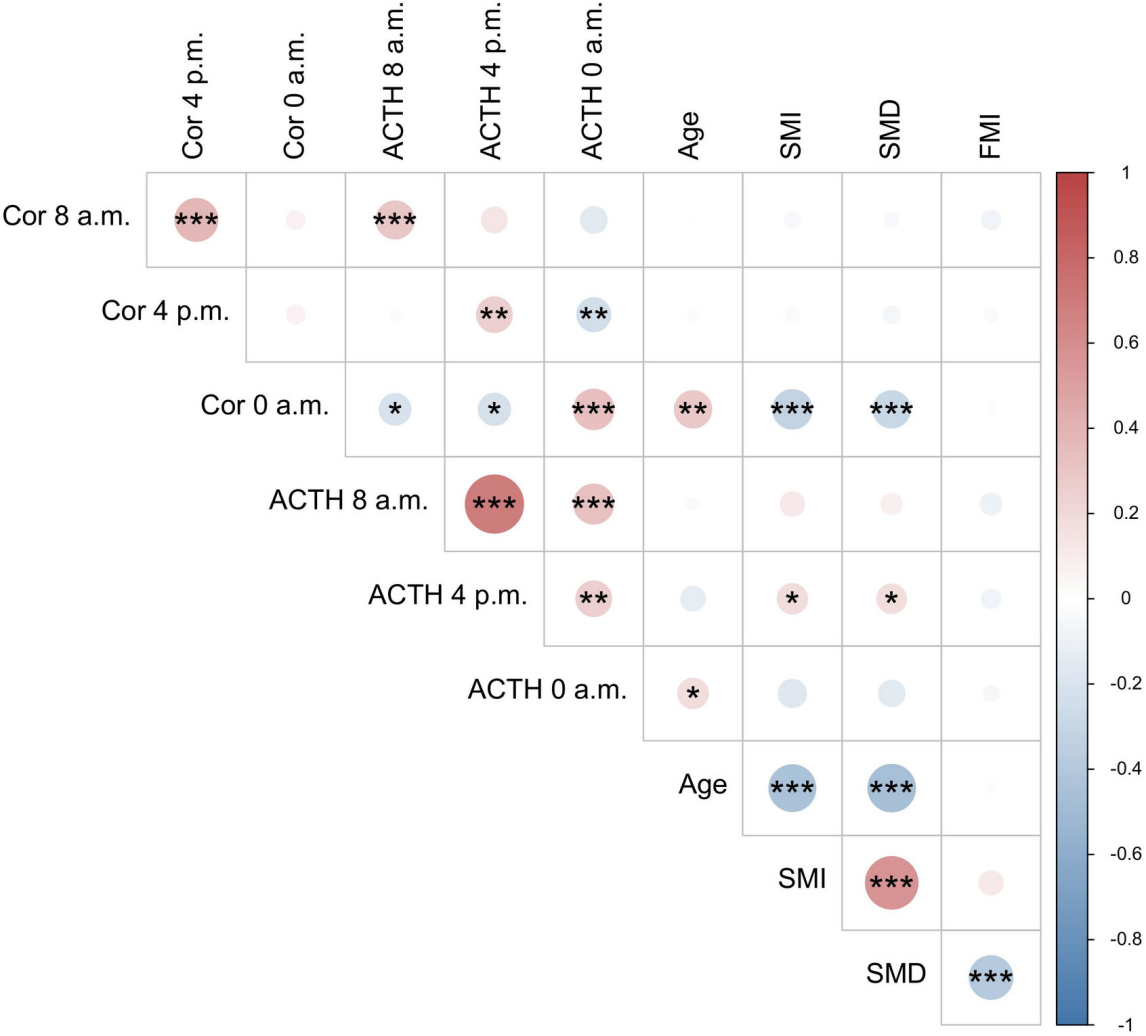
Correlations between cortisol, ACTH and body composition. Cor, cortisol. *p < 0.05; **p < 0.01; ***p < 0.001.

**FIGURE 3:**
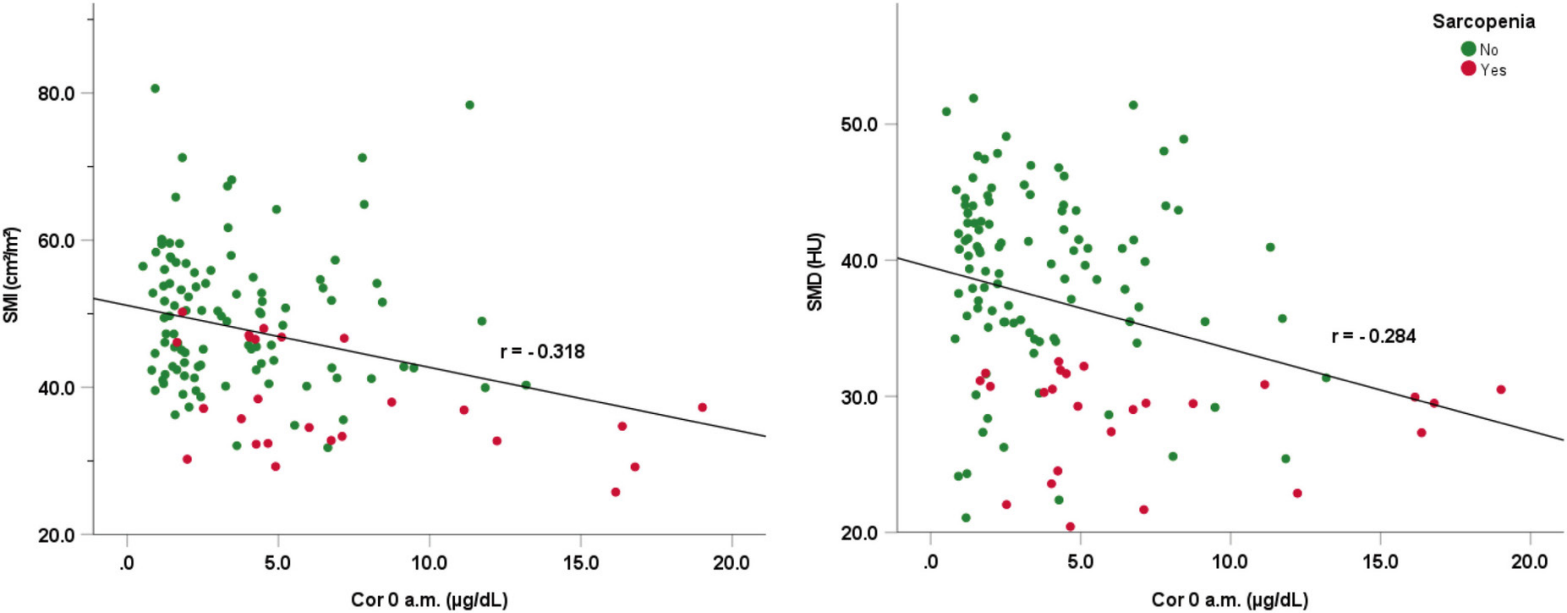
Scatter diagram of Cor 0 AM with SMI and SMD.

Correlations among body composition, age and cortisol rhythm are shown in Figure 2. Both Cor 0 AM and age were significantly negatively correlated with SMI (*r* = −0.318, *p* < 0.001 and −0.448, *p* < 0.001, respectively) and SMD (*r* = −0.284, *p* < 0.001 and −0.461, *p* < 0.001, respectively). Additionally, age was significantly positively correlated with Cor 0 AM (*r* = 0.283, *p* = 0.001) and ACTH at 0 AM (*r* = 0.188, *p* = 0.034). A scatter diagram of Cor 0 AM with SMI and SMD is shown in Figure 3. Weak but significant correlations between ACTH levels at 4 PM with SMI and SMD (*r* = 0.187, *p* < 0.05 and 0.176, *p* < 0.05, respectively) were noted.

### 3.3 Characteristics Based on Cor 0 AM Tertiles

The enrolled patients were divided into three groups based on their Cor 0 AM tertiles: low (T1), intermediate (T2) and high (T3) groups. Body composition and clinical characteristics are shown in Table 2. Participants of T3 group were significantly older and had lower levels of alanine transaminase, SMI and SMD.

**TABLE 2:** Characteristics according to Cor 0 AM tertiles.

Variables	Cor 0 AM tertiles			*p* value
	T1 (*n* = 43)	T2 (n = 43)	T3 (*n* = 42)	
Male (%)	32 (74.4)	29 (67.4)	22 (52.4)	0.095
Age (years)	48.58 ± 14.83	57.26 ± 10.21^a^	57.52 ± 13.39^a^	0.002
DM course (years)	6.00 (2.00, 11.50)	7.00 (3.00, 11.00)	8.00 (2.00, 17.00)	0.474
BMI (kg/m^2^)	26.99 ± 3.89	26.04 ± 3.31	25.38 ± 3.01	0.095
Haemoglobin (g/L)	144.12 ± 16.96	139.95 ± 14.04	137.12 ± 27.80	0.287
Creatinine (μmol/L)	62.06 ± 14.96	62.72 ± 16.31	59.19 ± 14.12	0.543
Blood urea nitrogen (mmol/L)	5.33 ± 1.62	5.50 ± 1.45	5.58 ± 1.60	0.758
TG (mmol/L)	1.44 (1.13, 2.25)	1.34 (0.92, 2.16)	1.18 (0.89, 1.81)	0.326
Total cholesterol (mmol/L)	4.67 ± 0.99	4.70 ± 1.29	4.61 ± 1.32	0.948
HDL (mmol/L)	1.15 ± 0.25	1.17 ± 0.24	1.17 ± 0.26	0.872
LDL (mmol/L)	2.87 ± 0.75	2.77 ± 1.05	2.86 ± 0.88	0.856
Albumin (g/L)	44.75 ± 4.58	42.49 ± 4.50^a^	44.35 ± 4.25	0.049
Alanine transaminase (U/L)	19.30 (13.90, 37.90)	17.20 (13.95, 31.20)	15.30 (11.70, 18.70)^a^ ^,^ ^b^	0.021
FBG (mmol/L)	7.87 ± 3.35	7.04 ± 1.71	8.19 ± 2.89	0.141
HbA1c (%)	8.69 ± 1.82	8.49 ± 2.07	9.24 ± 2.93	0.319
SBP (mmHg)	137.47 ± 20.30	137.93 ± 22.19	144.05 ± 22.09	0.295
DBP (mmHg)	86.74 ± 21.30	83.53 ± 16.00	80.48 ± 14.32	0.259
Dyslipidemia (%)	25 (58.1)	24 (55.8)	22 (52.4)	0.866
Hypertension (%)	27 (62.8)	30 (69.8)	26 (61.9)	0.706
Cigarette smoking (%)	16 (37.2)	18 (41.9)	14 (33.3)	0.718
Alcohol intake (%)	22 (51.2)	17 (39.5)	13 (31.0)	0.163
SMI (cm^2^/m^2^)	50.91 ± 9.19	47.50 ± 8.69	44.08 ± 11.53^a^	0.008
SMD (HU)	38.91 ± 7.07	37.05 ± 7.22	34.72 ± 7.52^a^	0.032
FMI (cm^2^/m^2^)	107.62 ± 41.75	112.52 ± 40.27	105.20 ± 33.32	0.674
Cor 8 AM (μg/dL)	11.15 (9.35, 14.01)	11.00 (9.87, 13.96)	10.97 (8.89, 15.01)	0.972
Cor 4 PM (μg/dL)	7.09 (4.79, 9.57)	5.86 (4.96, 7.41)	7.47 (5.66, 9.08)	0.102
Cor 0 AM (μg/dL)	1.41 (1.20, 1.65)^b^	3.32 (2.44, 4.14)^a^	7.03 (5.54, 9.48)^a^, ^b^	0.001
ACTH 8 AM (pmol/L)	5.62 (4.27, 7.96)	5.45 (3.94, 8.47)	4.18 (3.31, 6.76)	0.065
ACTH 4 PM (pmol/L)	4.39 (2.54, 5.39)	2.89 (2.09, 4.21)	3.02 (2.37, 4.00)	0.064
ACTH 0 AM (pmol/L)	1.80 (1.30, 2.29)	1.96 (1.31, 2.65)	2.87 (1.85, 4.21)^a^, ^b^	<0.001

Abbreviations: Cor, cortisol; DBP, diastolic blood pressure; DM, diabetes mellitus; FBG, fasting blood glucose; HDL, high‐density lipoproteins; LDL, low‐density lipoproteins; SBP, systolic blood pressure; TG, triglycerides.

^a^ Statistically significant difference with T1 group.

^b^ Statistically significant difference with T2 group.

### 3.4 Association Between Cor 0 AM and Sarcopenia

To assess the association between Cor 0 AM and sarcopenia, a cumulative logit model was applied, and the ORs for sarcopenia across the Cor 0 AM tertiles were calculated (Table 3). In the unadjusted model, the ORs for sarcopenia showed an increasing trend as the Cor 0 AM tertiles increased (OR = 4.69 [0.93, 23.53], *p* = 0.061 for T2 group and OR = 11.39 [2.41, 53.84], *p* = 0.002 for T3 group). After adjustment for multiple risk factors, the T3 group still had a significantly higher risk of sarcopenia than those in the T1 group (OR = 7.92 [1.45, 43.29], *p* = 0.017).

**TABLE 3:** ORs for sarcopenia according to Cor 0 AM tertiles.

Models	Cor 0 AM tertiles					*p* value
	T1 (*n* = 43)	T2 (n = 43)	*p* value	T3 (*n* = 42)	*p* value	
Sarcopenia, *N* (%)	2 (4.7)	8 (18.6)	0.044	15 (35.7)	< 0.001	0.001
Unadjusted	1	4.69 (0.93, 23.53)	0.061	11.39 (2.41, 53.84)	0.002	0.005
Model 1	1	3.71 (0.71, 19.32)	0.117	8.19 (1.66, 40.31)	0.009	0.025
Model 2	1	2.90 (0.50, 16.89)	0.237	7.92 (1.45, 43.29)	0.017	0.045

*Note:* Data are expressed as OR (95% CI). Model 1: adjusted for sex, age, haemoglobin and alanine transaminase; Model 2: Model 1 + DM course, BMI, creatinine, blood urea nitrogen, triglycerides, total cholesterol, high‐density lipoproteins, low‐density lipoproteins, albumin, fasting blood glucose, HbA1c, dyslipidemia, hypertension, cigarette smoking and alcohol intake.

Abbreviation: Cor, cortisol.

**FIGURE 4:**
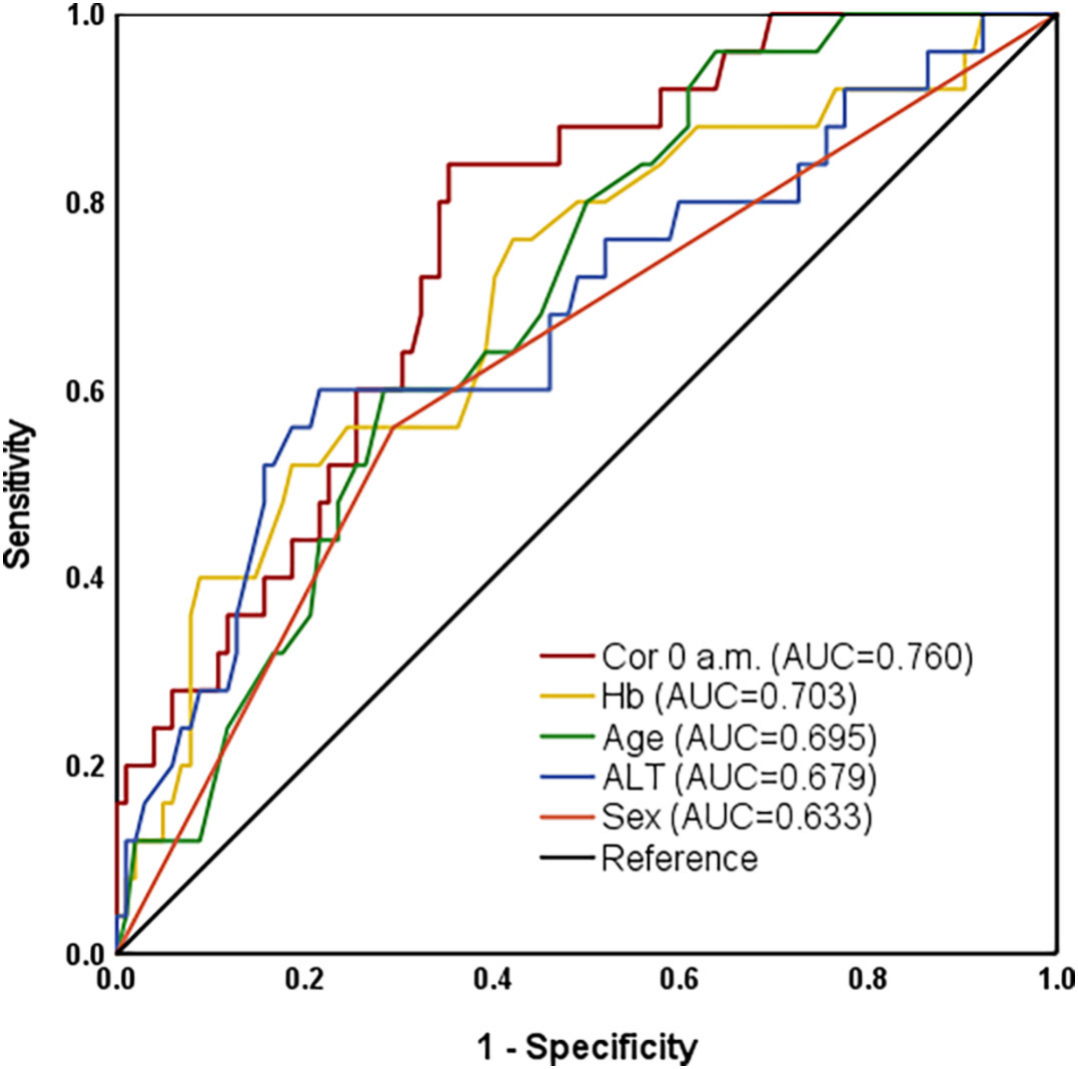
ROC analyses of risk factors for sarcopenia. ALT, alanine transaminase; Hb, haemoglobin.

Sex, age, haemoglobin, alanine transaminase and Cor 0 AM were variables with significant differences between the control and sarcopenia groups. Results of ROC curve analyses that compared their diagnostic performance for sarcopenia showed that Cor 0 AM had the highest predictive power (AUC = 0.760) compared with haemoglobin, age, alanine transaminase and sex (AUC = 0.703, 0.695, 0.679 and 0.633, respectively) (Figure 4).

## 4 Discussion

In this study, Cor 0 AM was negatively correlated with both SMI and SMD and was an independent risk factor for sarcopenia in individuals with T2DM. Higher levels of Cor 0 AM were associated with a higher risk of sarcopenia, and this association remained significant after adjusting for multiple risk factors. To the best of our knowledge, this is the first study to demonstrate a relationship between cortisol circadian rhythm and sarcopenia in patients with T2DM.

Sarcopenia was proposed as a clinical condition of substantial loss of muscle mass and function observed with ageing, which results in loss of independence among older adults [18]. One major cause of muscle mass and strength loss with ageing appears to be due to altered hormonal networks, including testosterone, oestrogen, cortisol, vitamin D, growth hormone, ghrelin and insulin [19]. Unlike most hormones that diminish throughout ageing, cortisol concentrations increase, displaying a flattened circadian profile with a higher evening nadir and an attenuated awakening response [20]. In our study, we found that ageing is associated with higher nighttime nadirs of cortisol, and both factors are linked to lower SMI and SMD. These results suggest that the circadian rhythm of cortisol during ageing is involved in the process of sarcopenia.

Glucocorticoids affect muscle mass through a combination of mechanisms that induce muscle proteolysis, decrease protein synthesis, and alter local production of growth factors that control muscle mass [21]. Several studies have investigated the relationship between cortisol and muscle mass or strength using various parameters, including salivary and serum cortisol, cortisol/DHEA ratio and 11β‐HSD1 expression [9, 22, 23, 24, 25]. Although most available evidence supports the involvement of cortisol alterations in sarcopenia, the association between serum cortisol concentrations and skeletal muscle mass was inconsistent [23, 25]. This study focused on the cortisol circadian rhythm and demonstrated that nighttime cortisol, rather than morning or afternoon cortisol, is significantly correlated with both SMI and SMD and is an independent risk factor for sarcopenia. Moreover, ROC curve analyses revealed that nighttime cortisol levels exhibit the highest predictive power for sarcopenia, underscoring its potential as a biomarker for diagnosing this condition. Furthermore, although not reaching statistical significance, patients with sarcopenia showed a trend towards lower ACTH levels at 8 AM, suggesting a diminished cortisol awakening response.

Diabetes mellitus is a complex metabolic disorder that affects different organs and leads to a series of concomitant diseases [26, 27, 28]. Interestingly, as a key player of circadian system, excessive cortisol plays an important role in the development of dyslipidemia, hypertension, cardiovascular disease and Alzheimer's Disease [29, 30, 31, 32]. Considering the elevated nighttime cortisol levels and flattened circadian rhythms in patients with T2DM [8, 10, 11], cortisol rhythm disorder might be a common cause of diabetic complications and comorbidities.

This study had several limitations. First, we did not assess muscle function (e.g., handgrip strength and gait speed), which has been proposed as one of the referred criteria for sarcopenia in the most recent consensus [12, 33, 34]. Further studies, including measurements of muscle function, are required to more definitively confirm the role of the cortisol circadian rhythm in sarcopenia. Second, as this was a retrospective study, other hormones that affect skeletal muscles, such as testosterone and growth hormone, were not included [7]. Third, the design of this study did not allow for causal inference and was limited to clarifying the underlying pathophysiological mechanisms. Thus, we could not determine whether the altered cortisol rhythm is a causative factor or a concomitant feature of sarcopenia. Finally, because our study was conducted on Chinese adults with T2DM, our findings may not be generalizable to other populations or ethnicities.

In conclusion, we demonstrated a significant association between cortisol circadian rhythm and sarcopenia in patients with T2DM. Patients with higher levels of nighttime cortisol, rather than morning or afternoon cortisol, have a higher risk of sarcopenia. This result offers a new strategy for the further research of sarcopenia.

## Ethics Statement

This study was approved by the Ethics Committee of the Affiliated Hospital of Jining Medical University (No. 2021C041). All patients were informed at admission that their medical records may be used for research purposes unless they indicated their opposition, and they provided written broad consent. For the present study, no patient indicated opposition.

## Conflicts of Interest

The authors declare no conflicts of interest.

## Data Availability

All data generated or analysed during this study are included in this published article. Further inquiries for the original data can be directed to the corresponding authors.
